# The Association between the Activin A Serum Level and Carotid Intima-Media Thickness in Chronic Kidney Disease Patients

**DOI:** 10.1155/2020/8893653

**Published:** 2020-11-09

**Authors:** Ade Yonata, Zulkhair Ali, Taufik Indrajaya, Erial Bahar, Ian Effendi, Novadian Suhaimi, Suprapti Suprapti

**Affiliations:** ^1^Department of Internal Medicine, Faculty of Medicine, Lampung University, Bandar Lampung 35141, Indonesia; ^2^Department of Internal Medicine, Faculty of Medicine, Sriwijaya University, Palembang 30126, Indonesia; ^3^Department of Anatomy, Faculty of Medicine, Sriwijaya University, Palembang 30126, Indonesia

## Abstract

**Introduction:**

Chronic kidney disease (CKD) is associated with high mortality rates, mainly as a result of cardiovascular complications. Meanwhile, recent studies have suggested a role of a homodimer protein called activin A in chronic kidney disease-mineral and bone disorder (CKD-MBD) conditions that may exist in the vascular calcification and osteolytic process. Ultrasound examination of the carotid intima-media thickness (cIMT) is a noninvasive method to assess vascular calcification. This study aimed to analyze the relationship between the activin A serum level and cIMT in patients with CKD at Mohammad Hoesin Hospital, Palembang, Indonesia.

**Methods:**

We conducted a hospital-based, cross-sectional study of consecutive CKD patients at the Department of Internal Medicine, Mohammad Hoesin Hospital, from July to November 2019. The level of activin A was measured by enzyme-linked immunosorbent assay. Meanwhile, cIMT measurements were collected by *B*-mode ultrasound imaging.

**Results:**

A total of 55 patients with CKD were included in this investigation. The median serum activin A level in these patients was 236.17 (116.33–283) pg/mL, while the median cIMT was 0.8 (0.6–1.45) mm. A relationship between the serum activin A level and cIMT (*r*  =  0.449; *p* = 0.001) was observed. During multivariate analysis with linear regression, triglyceride (*p* = 0.049), phosphate (*p* = 0.005), and activin A (*p* = 0.020) serum levels were factors associated with cIMT.

**Conclusion:**

In this study, a relationship between the activin A serum level and cIMT in patients with CKD was identified. Vascular calcification should be screened for in all CKD patients by the measurement of cIMT.

## 1. Introduction

Chronic kidney disease (CKD) is associated with high mortality rates attributed to the onset of cardiovascular complications. The risk of death exceeds that of type 2 diabetes mellitus and is linked to the mineral and bone disorders (MBD) that occur when suffering from CKD [1].

Vascular calcification falls under the umbrella of CKD-MBD [2]. Prior observational studies have shown this phenomenon occurs in 25% of patients with stage 3 CKD and in more than 50% of patients on dialysis [3]. Furthermore, it can be measured in various ways, including by an echocardiography examination to determine the carotid intima-media thickness (cIMT) [4].

The modern pathogenesis of CKD-MBD has grown increasingly complex as a deeper understanding of the concept of molecular level pathogenesis had been gained and discoveries of new biomolecules in the pathogenesis of CKD have been made. Several biomolecules such as Klotho, fibroblast growth factor-23 (FGF-23), and Wnt inhibitors are now recognized for their roles in the pathogenesis of CKD-MBD [5, 6]. Agapova et al. previously demonstrated activin A receptor signaling (ActRllA) signal activation using mouse CKD models [7]. ActRllA is a receptor for transforming growth factor-*β* protein family ligands [7, 8] whose activation enhances vascular calcification, while its signal inhibition restores conditions in the CKD model mice [7]. The study by Agapova et al. serves to increase the interest in researching the role of activin A as the most recent biomarker identified in CKD-MBD.

## 2. Materials and Methods

The present investigation was a hospital-based, cross-sectional consecutive study performed in the Department of Internal Medicine, Mohammad Hoesin Hospital, Palembang, Indonesia, from July to November 2019. Eligible patients were those older than 18 years with CKD diagnosed according to the National Kidney Foundation's Kidney Disease Outcomes Quality Initiative guidelines and who were willing to participate by signing an informed consent form. Meanwhile, patients with certain disorders such as rheumatoid arthritis, coronary bypass surgery or stent placement, inflammatory bowel disease, sepsis, and trauma capable of affecting the activin A level and/or cIMT diameter were excluded from this research.

Activin A levels were measured with the enzyme-linked immunosorbent assay sandwich technique using human activin A reagent from Cloud-Clone Corporation (Houston, TX, USA), while the cIMT examination was conducted using *B*-mode ultrasonography (Phillips Affinity 50) by a cardiovascular consultant. Furthermore, laboratory assessments such as urea, creatinine, calcium, phosphate, cholesterol, and parathyroid hormone levels were performed on the same day as activin A and cIMT tests.

This study was conducted in accordance with the principles of the Declaration of Helsinki. All protocols used throughout were approved by the Mohammad Hoesin Hospital Ethics Committee. Furthermore, all subjects provided informed consent and were given the opportunity to refuse participation.

Factors such as age, body mass index (BMI), activin A level, cIMT, urea, creatinine, phosphate, cholesterol, calcium, and parathyroid hormone are presented as mean ± standard deviation or median (min–max) values. Univariate analyses were performed using the chi-squared test and Student's *t*-test. Independent variables with *p* values of less than 0.25 in the analysis were also included into the multivariate linear regression analysis using a backward selection method. All statistical analyses were conducted using the Statistical Package for the Social Sciences version 19 (IBM Corporation, Armonk, NY, USA), where *p* < 0.05 was considered to be statistically significant.

## 3. Results

A total of 55 CKD patients were sampled, and their demographic and clinical characteristics are shown in Table 1. The median age of the study participants was 56 (range: 23–79) years. The study population included 37 (67.3%) men and 18 (32.7%) women with a duration of CKD ranging from one to 12 years. Moreover, the mean ± standard deviation BMI of the study sample was 22.8 ± 3.67. Twenty-five patients (45%) had stage 3 or 4 CKD, while 30 (55%) had stage 5 and were on dialysis. It was found that the median activin A level in the study population was 236.17 (116.33–283) pg/mL, while the mean cIMT was 0.8 (0.6–1.45) mm.

Notably, a correlation between the activin A level and a history of hypertension (*p* = 0.006) was observed (Table 2). The Spearman correlation analysis results also suggested the existence of a correlation between cIMT and triglycerides (*r* = 0.303; *p* = 0.025) or the phosphate serum level (*r* = 0.290; *p* = 0.032). Furthermore, a correlation was confirmed between the activin A serum level and the cIMT (*r* = 0.449; *p* = 0.001).

Variables with *p* < 0.25 in the univariate were further analyzed by multivariate analysis; subsequently, the results obtained from the multivariate analysis with linear regression showed that triglycerides (*p* = 0.049), phosphate (*p* = 0.005), and activin A (*p* = 0.020) serum levels were associated with the cIMT in the study population (Table 3).

## 4. Discussion

Among a total of 55 patients with CKD, 25 (45%) had stage 3 or 4 disease and 30 (55%) had stage 5 disease and were on dialysis. All 55 patients were in relatively stable condition without severe complications such as sepsis/severe infection, decompensated heart failure, cancer, or a severely complicated CKD-MBD condition.

The median level of activin A in the CKD patient sample was 236.17 (116.33–283) pg/mL. To date, studies of the activin A level have shown no agreement concerning the normal clinical serum range that should be adopted into daily practice. Instead, available data only suggest the optimal activin A levels in certain clinical conditions as compared with control group patients [9–11]. Use of the activin A level for clinical purposes is also still limited; instead, most studies in this area are focused on basic laboratory research. As such, the cost of an activin A test is rather expensive because it is still not widely used. Activin A could be used to explore female and male fertility [12, 13] and reproductive cancers [14]. High levels of activin A in serum have also been shown to be associated with tumor progression and poor prognosis in lung cancer [15]. The activin A level might also be used as a biomarker in patients with renal injury [16].

CKD-MBD starts early in CKD (stage 2), consisting of vascular osteoblastic calcification, osteodystrophy, and decreased klotho and increased FGF-23 secretion levels. Increased levels of activin A in CKD are relatively proportional to its expression in the kidneys. It is stimulated by factors that accompany chronic inflammation such as inflammatory cytokines and oxidative stress, which increase in CKD [17, 18].

Meanwhile, de Kretser et al. reported that the serum concentration of activin A was 0.11 ± 0.41 (0.036–0.283) ng/mL in patients with respiratory failure [9]. Furthermore, the level correlated with a significant increase in age, regardless of sex (*R*^2^ = 0.25*p* < 0.0001). Wu et al. conducted a research focusing on activin A levels in diabetes mellitus patients and revealed that the mean activin A levels in normal, impaired glucose tolerance, and diabetes patient groups were 90.1 ± 24.8 pg/mL, 89.6 ± 20.3 pg/mL, and 98.4 ± 31.6 pg/mL, respectively [10]. Elsewhere, Kuo et al. reported activin A levels in normal, prediabetes, and diabetes patient groups of 491.2 ± 165.3 pg/mL, 559.0 ± 178.5 pg/mL, and 572.7 ± 167.0 pg/mL, respectively [19]. Finally, research on activin A in patients with CKD undergoing hemodialysis was also carried out by Burowski et al. [11] and reported predialysis activin A levels of 122 ± 55.9 pg/mL.

Chhajed et al. observed cIMT in CKD patients to be 0.86 ± 0.21 mm vs. 0.63 ± 0.17 mm in the normal group [20]. Furthermore, Lahoti et al. found the diameter of cIMT in CKD patients to be 0.80 ± 0.28 mm. Lahoti analyzed cIMT diameter in each CKD stage but did not ascertain any correlation between the two (*r* = 0.119; *p* = 0.146) [21]. Moreover, the research conducted by Kumar et al. confirmed no correlation between the CKD stage and cIMT [22].

Not many studies to date have examined activin A levels in humans in association with clinical laboratory variables. Furthermore, the results we obtained were slightly different from those of Wu et al. who reported a correlation between the activin A level and BMI in patients with type 2 diabetes mellitus (*r* = 0.434) but no correlation in the normal patient or impaired glucose tolerance groups [10]. The lack of correlation between the activin A level and BMI in the latter groups may be due to the metabolic disorders and chronic inflammation in CKD that trigger increased levels of activin A, even in patients with low BMI values. In our study, there was no correlation between the serum activin A level and factors such as sex or serum cholesterol. These results are similar to those obtained by Wu et al. [10], while de Kretser et al. did not observe a significant effect of the activin A level on body weight, hip circumference, alcohol consumption, smoking, exercise, and allergies but noted a weak correlation between the activin A level and height (*R*^2^ = 0.031; *p* = 0.039), BMI (*R*^2^ = 0.028; *p* = 0.048), and waist circumference (*R*^2^ = 0.03; *p* = 0.042), respectively [9].

Of note, a history of hypertension was associated with the activin A level (*p* = 0.006) in our study. This result was also obtained by Wu et al. (*p* < 0.001) [10]. Furthermore, the research performed by Wu et al. found that serum levels of activin were positively correlated with age in the normal subjects group and the impaired fasting glucose group, while there was no positive correlation with age in the type 2 diabetes mellitus group. Our study also found no correlation between the activin A level (*p* = 0.053) and age. This may have occurred because some of our CKD patients were young, with glomerulonephritis since childhood.

Kuo et al. examined the levels of activin A in normal, prediabetes, and diabetes patients [19], reporting findings of 491.2 ± 165.3 pg/mL, 559.0 ± 178.5 pg/mL, and 572.7 ± 167.0 pg/mL, respectively. Furthermore, patients with prediabetes and diabetes had increased levels of activin as compared with that in the normal glycemic group. Wu et al. found no significant difference existed in activin A levels between the groups with the normal oral glucose tolerance test (OGTT), impaired OGTT, and type 2 diabetes (*p* > 0.05) [10]. In our study, levels of activin A had no correlation with a history of diabetes. Furthermore, variations in the inflammatory state between the groups studied may exist.

There are still not many studies covering the relationship between the activin A level and human cIMT, although Kuo et al. reported a relationship between the activin A level and cIMT in humans in a population of prediabetes and diabetes patients [19]. The results obtained through Spearman regression logistic analysis of the total population (*N* = 457) suggested a positive correlation between the activin A level and cIMT (*r* = 0.263; *p* < 0.001). However, Kuo et al. reported that the activin A level was positively correlated with cIMT in the prediabetes group (*r* = 0.264, *p* = 0.001) but not so in the diabetic group. These results were similar to those obtained in our study, where the activin A level was positively correlated with cIMT in patients with CKD. Until now, there has been no research suggesting the relationship between the activin A level and cIMT in patients with CKD.

cIMT thickening involves many processes that contribute to arteriosclerosis and vascular calcification [23–25]. Agapova et al. reported an increase in activin A circulation (by peritubular myofibroblast) in CKD patients [7] and also showed the role of ActRIIA in vascular lesions caused by CKD using mouse models. Furthermore, the ActRIIA ligand trap (activin A receptor blocking ligand) RAP-011 given in the CKD mouse model triggered a decrease in the activin A level, increased the contractile effect of vascular smooth muscle, increased the expression of cell-specific proteins in the aorta, reduced the osteoblast count, and lessened the degree of atherosclerotic intimal calcification. In CKD, increased messenger RNA expression of osteogenic proteins such as runx2 and alkaline phosphatase has been reported, leading to increased vascular calcification [7, 26, 27]. The administration of RAP-011 and ActRllA ligand trap, which decreases activin A levels, can apparently inhibit and improve this condition [7]. The research performed by Agapova et al. reports an increase in activin A levels in CKD conditions and the role it plays in increasing vascular calcification in a mouse model [7].

Activin A levels are known to increase in CKD [7, 28], and its mechanism in vascular calcification might act via ActRllA receptor signaling [7]. The binding of activin A with this receptor will produce an intracellular activin A signal that activates smad2 phosphorylation through the RANKL pathway that forms the smad2 complex [29, 30]. In this context, acetylation by CBP protein to smad2 occurs, which causes translocation of the smad2 complex from the cytosol into the nucleus [18, 29]. The smad2/cfos complex then becomes attached to the promoter region of the gene that plays a role in vascular calcification [29]. Furthermore, the vascular calcification process can be measured by assessing the cIMT using *B*-mode ultrasonography. This is the first investigation to show the association between activin A and cIMT in CKD. The results obtained herein highlighted a correlation between the activin A level and cIMT in CKD patients. Therefore, our findings support the theory of the role of activin A in the process of vascular calcification in patients with CKD.

## 5. Conclusions

Activin A serum level was associated in the multivariate logistic regression model with cIMT in CKD patients. Furthermore, studies are required to clarify this association, including revealing related molecular and biological mechanisms. Furthermore, vascular calcification should be screened for in all CKD patients by the measurement of cIMT.

## Figures and Tables

**Table 1:** Demographic and clinical characteristics of the study population.

Characteristic	Sample, *N* = 55

Age	56 (23–79)
Sex	
Male	37 (67.3%)
Female	18 (32.7)
CKD duration (years)	1 (1–12)
BMI	22.8 ± 3.67
CKD etiology	
Hypertension	19 (34.5%)
Diabetes	15 (27.3%)
Obstructive uropathy	3 (5.5%)
Glomerulonephritis	6 (10.9%)
CKD stage	
3-4	25 (45%)
5 on dialysis	30 (55%)
Urea (mg/dL)	77.7 ± 36.5
Creatinine (mg/dL)	3.93 (1.51–15.9)
LDL (mg/dL)	100 ± 41.1
Triglycerides (mg/dL)	140.1 ± 66.4
CRP (mg/dL)	10 (4–230)
Phosphate (mg/dL)	3.7 (1.5–10.5)
Calcium (mg/dL)	9 (7–118)
Albumin (mg/dL)	3.8 (1.9–6.8)
PTH (pg/mL)	36.4 (6.8–1251.2)
Activin A (pg/mL)	236.17 (116.33–283)
cIMT (mm)	0.8 (0.6–1.45)

BMI, body mass index; CKD, chronic kidney disease; CRP, C-reactive protein; LDL, low-density lipoprotein; PTH, parathyroid hormone

**Table 2:** Association of risk factors and the activin A serum level with cIMT.

Risk factor	Activin A		cIMT	
	*r* value	*p* value	*r* value	*p* value
Age	−0.263	0.053	−0.149	0.278
Sex	—	0.739^*a*^	—	0.977^*a*^
CKD duration	0.227	0.096	0.250	0.066
BMI	0.213	0.118	0.104	0.450
DM	—	0.282^*a*^	—	0.063^*a*^
Hypertension	—	0.006^*∗*^	—	0.801^a^
LDL (mg/dL)	−0.137	0.318	−0.099	0.472
Triglycerides (mg/dL)	0.038	0.782	0.303	0.025^*∗*^
CRP (mg/dL)	−0.249	0.066	−0.243	0.074
Phosphate (mg/dL)	0.205	0.134	0.290	0.032^*∗*^
Calcium (mg/dL)	0.075	0.588	0.010	0.943
Albumin (mg/dL)	0.106	0.443	−0.065	0.638
PTH (pg/mL)	0.230	0.092	0.183	0.180
Activin A (pg/mL)	—	—	0.449	0.001^*∗*^

BMI, body mass index; CKD, chronic kidney disease; CRP, *C*-reactive protein; DM, diabetes mellitus; LDL, low-density lipoprotein; PTH, parathyroid hormone. ^*∗*^Statistically significant values.

**Table 3:** Multivariate linear regression analysis.

Model	Unstandardized coefficient		Standardized coefficient	*T*	Sig.
	*B*	Std. error	Beta		
Constant	0.043	0.01		4.222	0.000
Phosphate	0.004	0.001	0.351	2.903	0.005
Triglycerides	6.816E-5	0.000	0.235	2.017	0.049
Activin A	9.722E-5	0.000	0.284	2.399	0.020

## Data Availability

The SPSS research data used to support the findings of this study are available from the corresponding author upon request.
